# Study of graphene p-n junctions formed by the electrostatic modification of the SiO_2_ substrate

**DOI:** 10.1038/s41598-024-61683-2

**Published:** 2024-05-28

**Authors:** Tharanga R. Nanayakkara, U. Kushan Wijewardena, Annika Kriisa, Ramesh G. Mani

**Affiliations:** 1 Georgia State University, Atlanta, GA 30303 USA; 2 Brookhaven National Laboratory, Upton, NY 11973 USA; 3 Georgia College and State University, Milledgeville, GA 31061 USA

**Keywords:** Condensed-matter physics, Electronics, photonics and device physics, Materials science, Physics

## Abstract

We study the transport properties of mm-scale CVD graphene p-n junctions, which are formed in a single gated graphene field effect transistor configuration. Here, an electrical-stressing-voltage technique served to modify the electrostatic potential in the SiO_2_/Si substrate and create the p-n junction. We examine the transport characteristics about the Dirac points that are localized in the perturbed and unperturbed regions in the graphene channel and note the quantitative differences in the Hall effect between the perturbed and unperturbed regions. The results also show that the longitudinal resistance is highly sensitive to the external magnetic field when the Hall bar device operates as a p-n junction.

## Introduction

Graphene^1,2^ denotes a single layer of carbon atoms arranged in a honeycomb structure. This material has attracted considerable theoretical and experimental interest due to its extraordinary characteristics^3–6^. The Fermi level of pristine graphene lies at the Dirac or charge neutrality point. Yet, a feature of graphene is that the Fermi level can be swept across the Dirac point into conduction or valence band by doping or applying an external gate voltage. For pristine graphene, one expects a V-shaped conductance vs. gate voltage trace. In non-ideal graphene, the shape of the characteristic curve as well as the conductance value and the location of the Dirac or charge neutrality point provides useful information regarding the carrier mobility, the asymmetry between electron- and hole-conduction, the doping level, etc^2,7,8^. The V-shaped conductivity vs gate voltage curve, which implies a single Dirac point, has been widely observed in experimental studies of graphene. More rarely observed are unusual W-shaped conductivity vs. gate voltage curves, which imply double neutrality points in single-gated graphene field effect transistors that were not subjected to special device treatments.

Double neutrality points in a single device are a signature of the existence of p-n junctions in graphene. Such p-n junctions can be realized by using multiple gates in a single device to tune the channel to n-p-n, p-n-p, n-n′-n and p-p′-p type junctions. The device then shows the double neutrality points for a certain range of parameters^9–11^. Double neutrality points can also be exhibited in p-n junctions created by separate chemical doping^12–14^. Another approach to realizing double neutrality points utilizes the invasive nature of contacts^15,16^. These methods mentioned above require additional or special sample preparation steps, which might increase fabrication complexity for practical real-world applications. In a single gated, monolayer exfoliated graphene on SiO_2_ substrate, Chiu et al.^17^ have reported a novel electrical-stressing-voltage approach to create p-n junctions , which changes the local electrostatic potential with out using multiple gates. This approach included the advantage that the electronic modification occurs in the substrate, but not in the graphene film. Yu et al.^18^ also reported the formation of p-n junction in graphene via this electrical stressing-voltage-method and they reported that mobility improvement is realized by reducing the impurity induced scattering in graphene as a result of low level stressing voltage^18^. Therefore, this stressing-voltage-technique has also been applied to improve the quality of graphene based devices^19,20^.

Here, we report an investigation of double neutrality points formed by applying an electrical-stressing-voltage^17,18^ on millimeter sized CVD graphene specimens. As in the earlier studies, the electrical-stressing-voltage appears to modify the electronic characteristics of the SiO_2_ substrate and the conductivity in graphene^17,18^. To gain quantitative insight into the channel characteristics, we modeled and extracted the mobility and residual carrier concentrations around the neutrality points^17^. The results indicate a mobility of 6148 $\text{cm}^2/Vs$ in the unperturbed region in comparison to a mobility of 1388 $\text{cm}^2/Vs$ in the perturbed region. A comparative study of the Hall effect in the unperturbed and perturbed region yields a current related maximum sensitivity of 2635 V/AT. When a backgate voltage biases the electronic system between the two neutrality points, which corresponds p-n junction in the channel, the longitudinal or diagonal resistance shows enhanced sensitivity to the external perpendicular magnetic field as the longitudinal or diagonal voltages obtain a Hall like component. Such electrostatic modification of the substrate can be exploited to create an abrupt p-n junction, which can be useful for engineering planar electronic Veselago lenses and focused beam splitters using controllable transistors based on graphene^21^. These results help to understand the intrinsic behavior of double neutrality points and p-n junctions in graphene field effect transistors, while contributing to a better awareness of the charge carrier characteristics in graphene p-n junctions.

## Experiment

**Figure 1:**
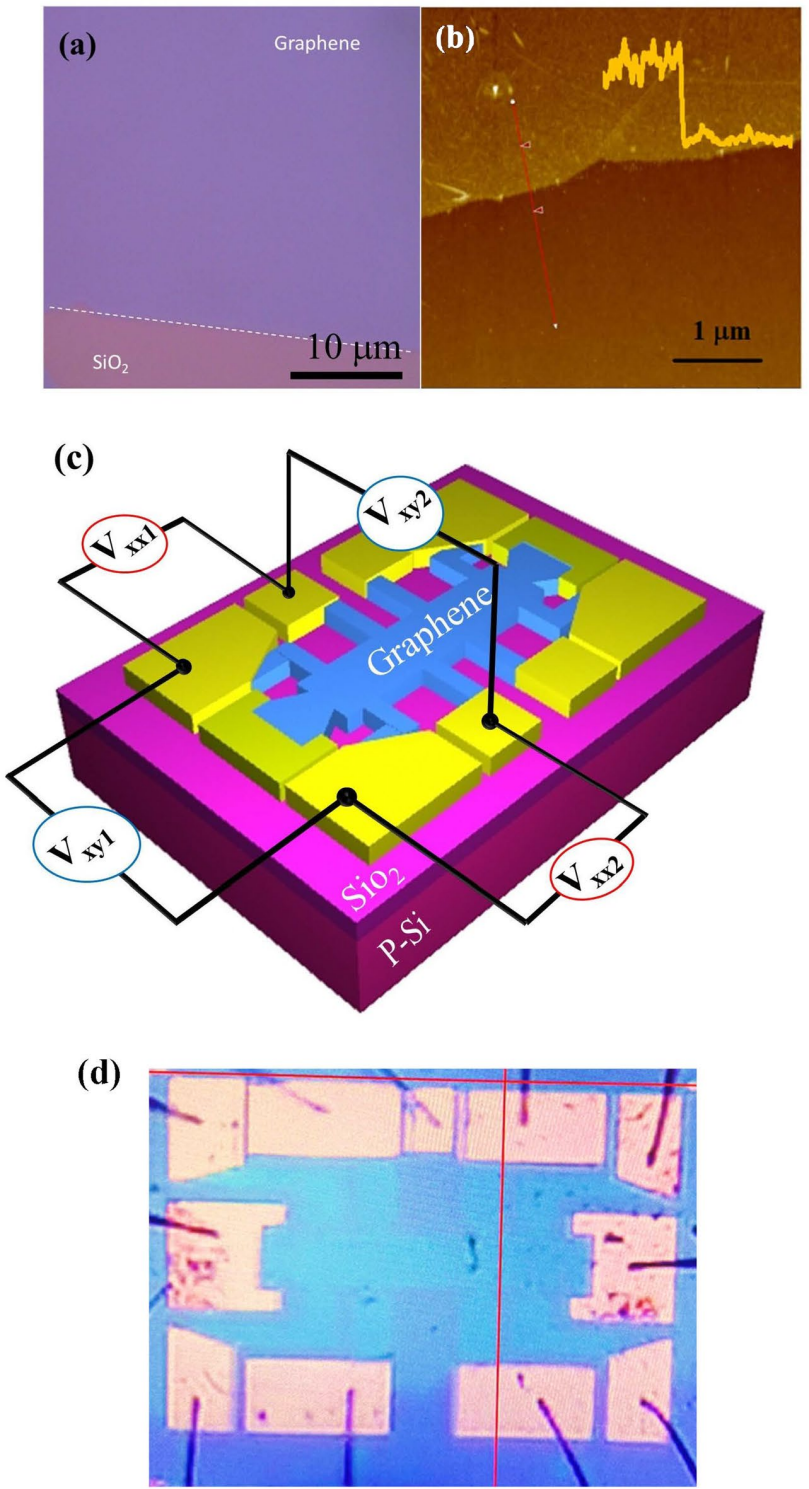
(**a**) An optical image of single layer graphene on the SiO_2_/Si substrate. The white dotted line shows the boundary between SiO_2_ and graphene. (**b**) AFM topographical image of the graphene on top of the SiO_2_. Inset shows the height profile along the red line. (**c**) A schematic diagram of the Hall bar device. Here, $V_{xx1}$ and $V_{xx2}$ are the longitudinal voltages, and $V_{xy1}$ and $V_{xy2}$ are the Hall voltages examined in this study. (**d**) A photograph of a measured device.

Chemical vapor deposition (CVD) graphene^22,23^ films were fabricated into millimeter-scale Hall effect devices using wet transfer techniques and photolithography, and four-terminal DC electrical measurements were carried out in vacuum. Color contrast of graphene on 300 nm thick SiO_2_ on p-Si confirmed monolayers, which was consistent with height profile ($\sim$1 nm) observed in atomic force microscope (AFM) measurements, see Fig. 1a and b, respectively. A sketch of the fabricated Hall bar device with the pin configuration is shown in Fig. 1c and the reported measurements are as labelled in this figure. Figure 1d shows a photograph of the device. Here, the p-Si-substrate layer served as a back-gate to control carrier density in the graphene layer and the back gate voltage sweep range was limited to $-50 \le V_{G} \le +50\; \text{V}$. Under ambient conditions, the gate voltage dependence of the diagonal resistance, $R_{xx}$, on freshly prepared graphene Hall bar sample at the room temperature exhibited strong hysteretic behavior. The observed behavior of the sample is partly attributed to dipolar adsorbates such as water, oxygen and poly-methyl-methacrylate (PMMA), which act as charge carrier traps. Charge carrier trapping by water is a dynamic process that depends on the sweep conditions of the back gate voltage^8^, as also observed in our measurements.

**Figure 2:**
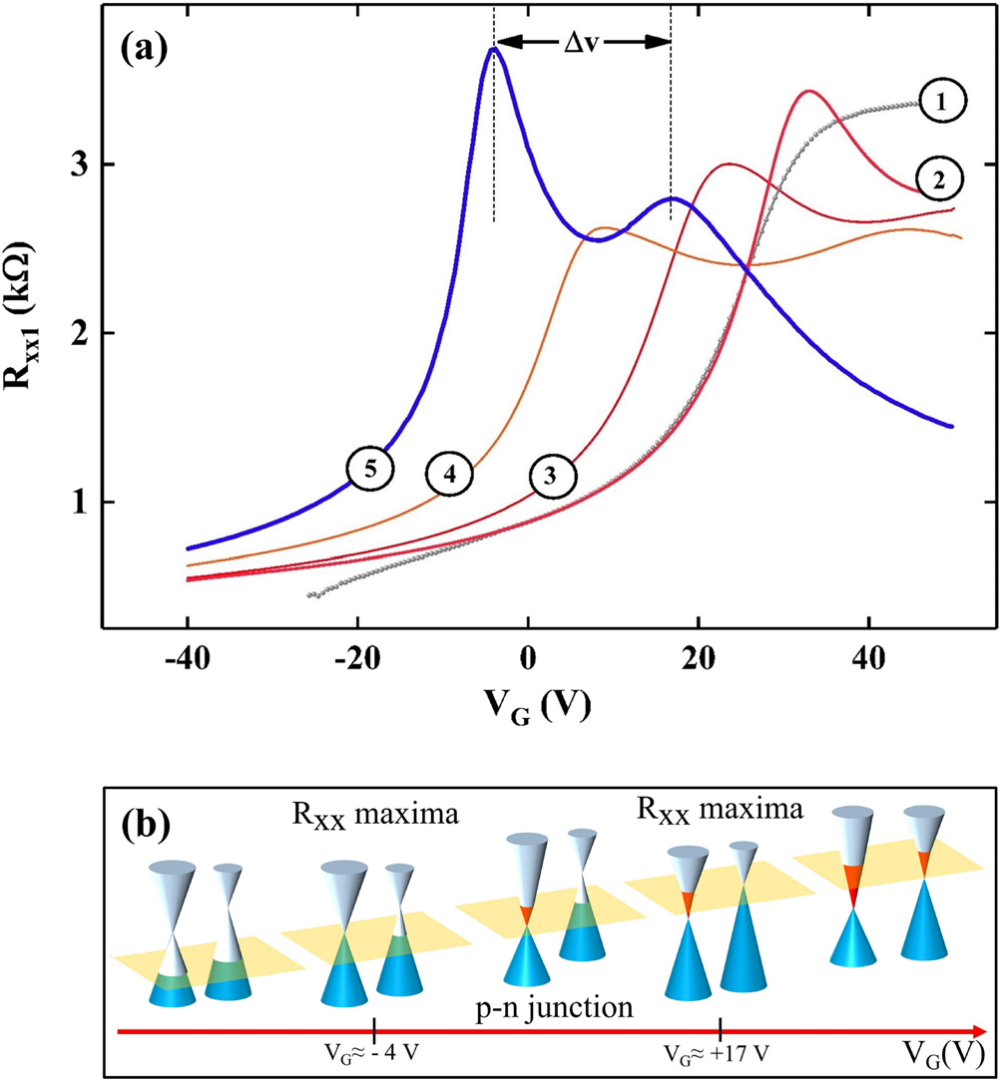
Inducing a p-n junction in a graphene device. (**a**) this panel illustrates the electrical stressing procedure that changes the $R_{xx1}$ vs. $V_{G}$ characteristics from (1) $\rightarrow$ (5), and brings about the formation of the p-n junction, as indicated by the twin peaks in (5). In (5), the $R_{xx1}$ exhibits maxima at $V_{G} \approx -4\; \text{V}$ and $V_{G} \approx +17 \; \text{V}$, as $\Delta v \approx 21 V$ is the back gate voltage difference between the peaks. (**b**) Tuning of the Fermi level in an electrically stressed graphene device by varying the back gate voltage can allow for the emergence of a p-n junction. Here, the two cones characterize two regions in graphene, and the yellow plane represents the Fermi level in the device.

In order to reduce trapped charge due to adsorbates on the surface, we stored the freshly prepared sample under vacuum at room temperature overnight. Such storage reduced the hysteretic behavior significantly and the charge neutrality point fell within the swept $V_{G}$ span. To introduce the second charge neutrality point, we applied electrical-stressing-voltages to the samples at room temperature with the sample in vacuum. As indicated in the literature, the electrical stressing method involves applying a large source drain voltage, together with a gate voltage on the specimen^17,18^. Here, instead of a large source-drain voltage, we applied a large current. Figure 2a illustrates traces obtained through a typical stressing procedure. Curve (1) in panel Fig. 2a displays $R_{xx1}$ as a function of gate voltage $V_{G}$ under ambient conditions. After the sample space was evacuated, the first neutrality point appeared here around $V_{G} = +33\; \text{V}$, as depicted by curve (2) in panel Fig. 2a. Curve (3) illustrates $R_{xx1}$ vs. $V_{G}$ after the sample was annealed with $I_{DC} = 1 \; \text{mA}$ for 30 min at room temperature in vacuum. This treatment shifted the first charge neutrality point to + 23 V, and indications of a second neutrality point emerged at the higher end of the gate voltage range. Subsequently, the sample underwent another annealing with 1 mA current for 60 min, with $V_{G} =-40 \; \text{V}$. This process moved the first neutrality point closer to $V_{G} =+8 \; \text{V}$, and the second neutrality point appeared around $V_{G} = +45 \; \text{V}$, as shown in curve (4) in panel Fig. 2a. Finally, the sample was cooled to approximately 15 K with a gate voltage of − 30 V, without applying an annealing current. Then, trace (5) in Fig. 2a was collected, which shows double neutrality points in the graphene device. We note that the second charge neutrality point disappeared when the device was exposed to ambient conditions for roughly two hours. Then, the specimen reverted to its initial state, but the p-n junction could be reestablished easily by reapplying the electrical stressing procedure. These repeatable and reversible features confirmed that the applied electrical stressing voltage did not irreversibly damage the device.

## Results and discussion

The double neutrality points observed in the $R_{xx}$ vs. the gate voltage traces are a signature of the formation of a p-n junction in the graphene channel. The schematic in Fig. 2b depicts the realization of the p-n junction in the graphene channel over a span that includes $-4 \le V_{G} \le 17$V. Two cones represent the graphene dispersion in two regions, and yellow plane represent the Fermi level of the system. When gate voltage is equal to − 50 V, the Fermi level lies below the both neutrality points, and the entire channel is p-type as in Fig. 2b. When the gate voltage is swept towards postive voltages from − 50 V , the Fermi level is shifted up and crosses the first neutrality point at $V_{G}\approx -4$ V, as $R_{xx1}$ (see Fig. 2a, trace (5)) increases and crosses its first maximum with the gate voltage increment. A further increase in $V_{G}$ progressively moves up the Fermi level and, when $V_{G}\approx +17$V, the Fermi level crosses the second neutrality point and this is reflected as the second peak in $R_{xx1}$ (see Fig. 2a, trace (5)). Hence, over the gate voltage span $-4<V_{G}<+17$ V, the sample includes a p-n junction. For $V_{G} > 17$V, the entire channel is n-type because the Fermi level lies in the conduction band throughout the channel.

To extract further information, we applied Eq. (1), the extended fitting equation for double neutrality points^24^, which assumes the existence of multiple parallel channels contributing to the current, with each channel characterized by its own value of the neutrality point.

1 $$\begin{aligned} R_{xx}= \frac{(1-\lambda )}{e\mu _{1} \sqrt{n_{01}^2+n_{1}^2}}+\frac{\lambda }{e\mu _{2} \sqrt{n_{02}^2+n_{2}^2}} +r_{s} \end{aligned}$$

Here, $\lambda (=0.63)$ is a parameter related to a geometrical factor in the graphene channel and that depends on the physical locations of the neutrality points. Also, $r_{s}$ is the resistance due to short range scatters, assumed to be gate independent^24^. We found that the $r_{s}$ can be neglected from the fittings of our results. The $\mu _{1}$ and $\mu _{2}$ are charge carrier mobilities and $n_{1}$ and $n_{2}$ are residual charge concentrations in the unperturbed- and perturbed-regions, respectively, in the graphene channel. The carrier densities in these two regions are determined by electrostatic potentials. Data fits indicated that the mobility values are 6148 $\text{cm}^2/Vs$ and 1388 $\text{cm}^2/Vs$ in unperturbed and perturbed regions of the graphene, respectively. Also, the extracted values for the residual carrier concentrations are $1.59\times 10^{11} \; \text{cm}^{-2}$ and $1.11\times 10^{12} \; \text{cm}^{-2}$ in unperturbed and perturbed regions of the graphene, respectively. Since it is known that as the carrier density increases, the mobility decreases for single layer graphene^25^, the high density of residual carriers in perturbed region could be responsible for a corresponding lower mobility. The application of a small perpendicular magnetic field, within the range $-0.2<B<+0.2$ T produced a small change in the $\Delta v$, where $\Delta v$ is the difference of the back gate voltage between resistance peaks. Further, $\Delta v$ is associated with a trapped charge concentration $n_{t}=\alpha \Delta v$ with $\alpha =7.2\times 10^{10} \; \text{cm}^{-2}\,\text{V}^{-1}$.

**Figure 3:**
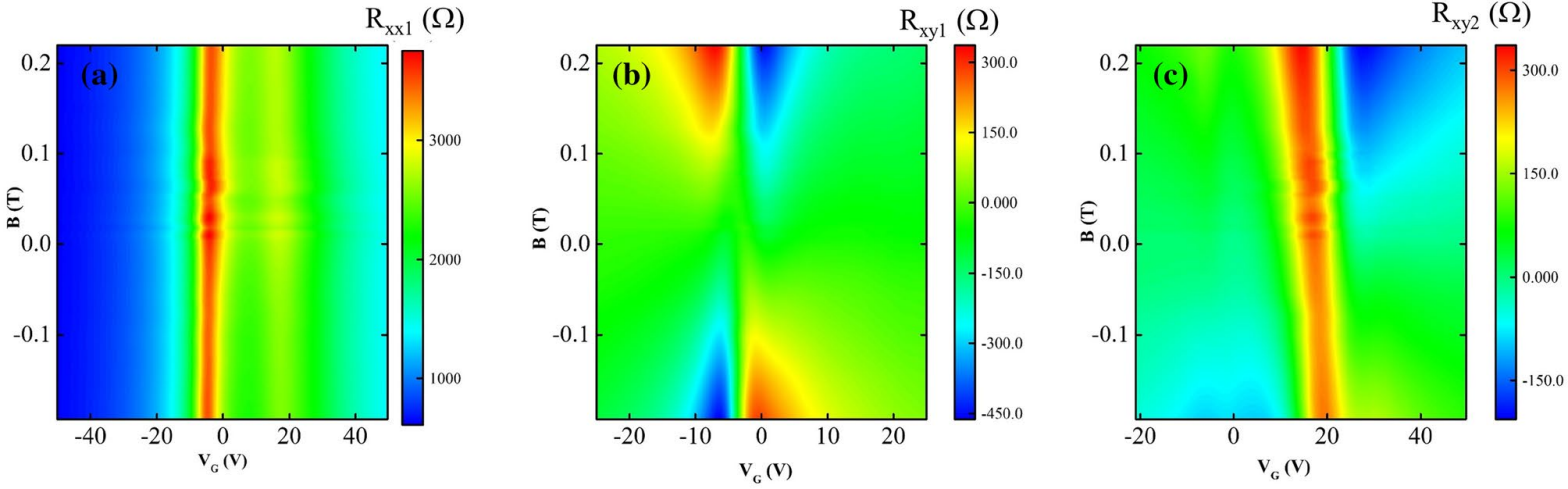
The longitudinal resistance ($R_{xx}$) and Hall resistances ($R_{xy}$) color maps for a Hall bar sample with double neutrality points, as function of back gate voltage ($V_{G}$) and magnetic field (*B*). (**a**) Shows the $R_{xx1}(B,V_{G})$. The $R_{xx1}(B,V_{G})$ plot exhibits maxima near two distinct charge neutrality points, see also Fig. 2b. (**b**) Shows the $R_{xy1}(B,V_{G})$ from the unperturbed region (see text) of the graphene channel. (**c**) Shows the $R_{xy2}(B,V_{G})$ from the perturbed region (see text) of the graphene channel.

The Fig. 3a shows the longitudinal resistance ($R_{xx1}$) as a function of magnetic field and gate voltage. The $R_{xx1}$ color map shows maxima at the charge neutrality points. An asymmetry between hole side and electron side of the characteristic curve is observable relative to the first charge neutrality point. The second charge neutrality point on the side of positive gate voltage may lead to this $R_{xx1}$ asymmetry around the original neutral point. Also, there was no significant difference in longitudinal voltage measurements $V_{xx1}$ and $V_{xx2}$ (see Fig. 1c) from two parallel sides of the Hall bar. The Fig. 3b represents the Hall resistance $R_{xy1}$ in the unperturbed region of the graphene channel, which is physically away from the second charge neutrality point. Figure 3c depicts the Hall resistance $R_{xy2}$ in the perturbed area of the graphene sample, which is physically close to the second neutrality point. Here, “unperturbed” and “perturbed” refer to the effect of the stress-voltage on the neutrality point.

**Figure 4:**
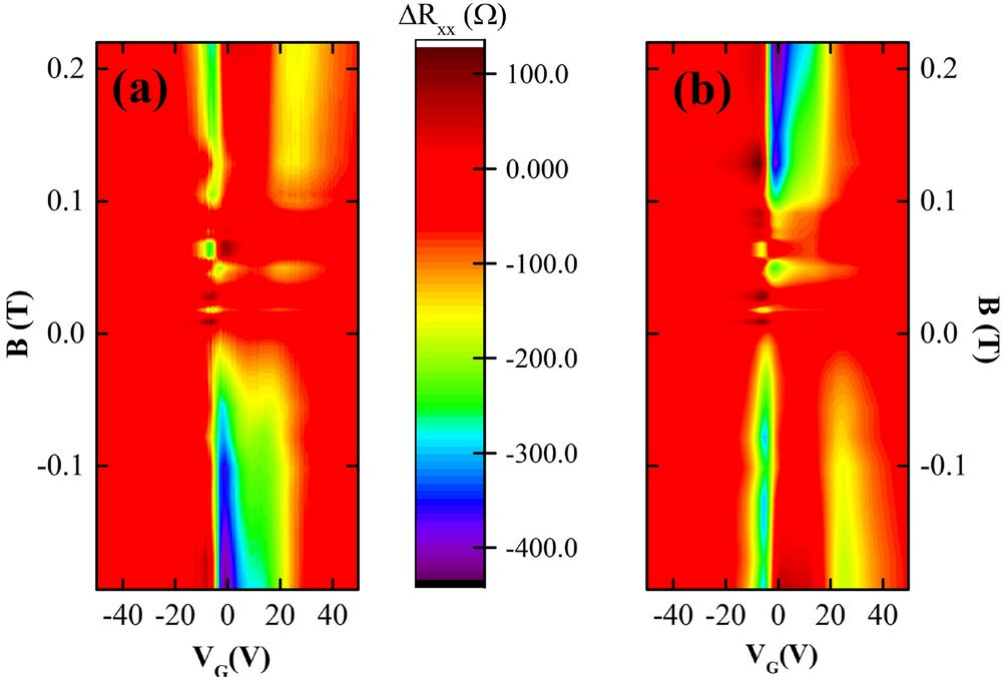
Here, (**a**) and (**b**) are color maps of $\Delta R_{xx}$ on the two sides (see Fig. 1c) of the Hall bar device as functions of the magnetic field (B) and the back gate voltage ($V_{G}$). Here, $\Delta R_{xx}(B,V_{G})=R_{xx}(B,V_{G})-R_{xx}(B=0,V_{G})$. $\Delta R_{xx}$ is more sensitive to the magnetic field at $V_{G}$ values in between the voltages corresponding to two neutrality points ($V_{D1}<V_{G}<V_{D2}$). (a) shows the $\Delta R_{xx}$ values from one side of the Hall bar, and (**b**) exhibits the $\Delta R_{xx}$ values from the other side (opposite to (**a**)) of the Hall bar.

Figure 4 shows the $\Delta R_{xx}=R_{xx(B\ne 0)}-R_{xx(B=0)}$ as a function of magnetic field and gate voltage, obtained from the two parallel sides of the Hall bar. The $\Delta R_{xx}$ values are close to zero over a large fraction of the color maps except where $V_{G}$ corresponds to gate voltages between two neutrality points. At the same time, the non-vanishing response depends on both the magnitude and the polarity of external magnetic field, with the two sides of the device showing similar behavior at opposite *B* field polarities. The latter feature can be understood from the observation that a non uniform Hall voltage between the perturbed and unperturbed regions must lead to an antisymmetric in *B* component in the longitudinal voltages, with polarity reversal on the two sides. The color plots also show a small response just beyond the two neutrality points to the opposite polarity of magnetic field in each side as shown in Fig. 4a and b.

**Figure 5:**
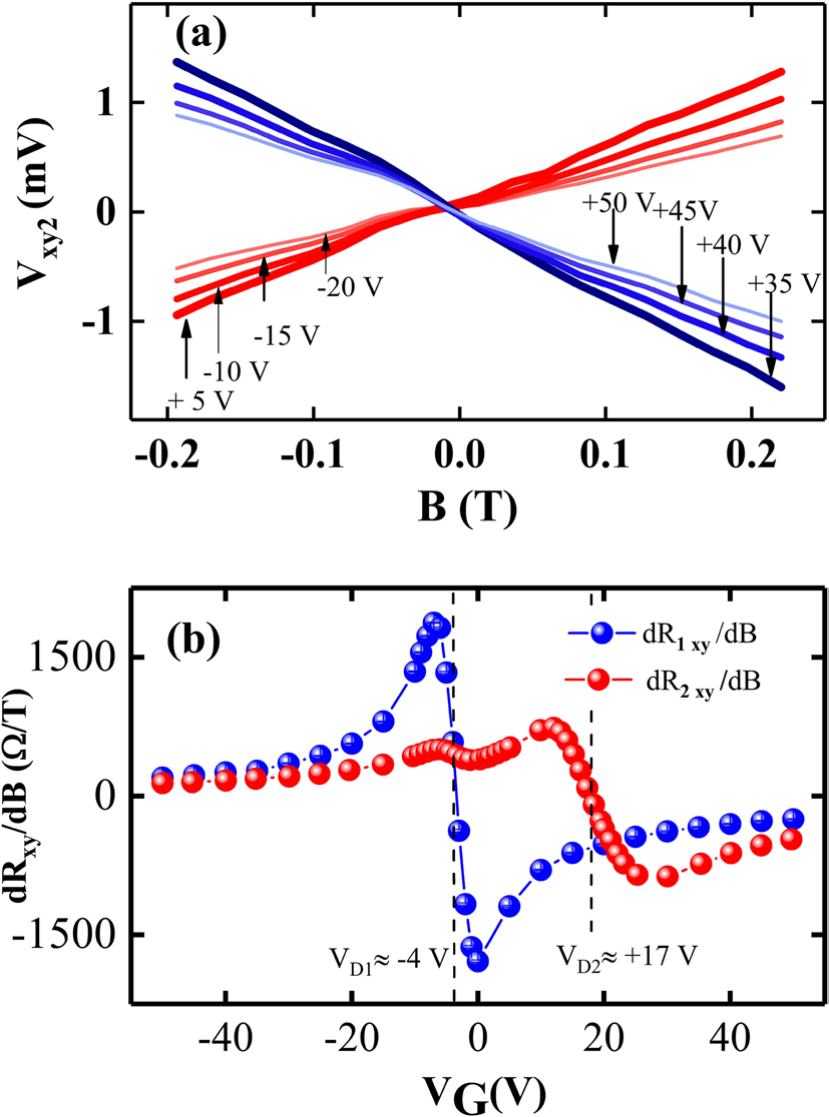
(**a**) The Hall voltage in the perturbed region ($V_{xy2}$) is plotted vs *B* with the back gate voltage ($V_{G}$) as the parameter. The red traces display a positive slopes that increases with $V_{G}$, while the blue lines exhibit a negative slope that decreases with $V_{G}$. (**b**) Typical variation in $dR_{xy}/dB$ vs $V_{G}$. The blue spheres show the extracted $dR_{xy}/dB$ values from the data in the unperturbed region of the graphene channel. The red spheres represent the extracted $dR_{xy}/dB$ values from the data in perturbed area of the graphene channel. The $dR_{xy}/dB$ represents the slope of $V_{xy}$ vs *B* graphs.

Figure 5a shows the Hall voltage with positive and negative slopes in the perturbed region of the graphene channel for selected back gate voltage values. Figure 5b presents the non-monotonic behavior in the slope of Hall voltages across the perturbed and unperturbed region of graphene channel, as it summarizes the slope of Hall resistances ($dR_{xy}/dB$) as a function of gate voltage. Blue spheres shows the Hall slope variation across the unperturbed region of the graphene channel. From − 50$<V_{G}<$− 5 V, the slope remains positive and increase in magnitude, then it starts decreasing from positive values to negative values within the gate voltage range − 5$<V_{G}<$0 V. From 0$<V_{G}<$+ 50 V, Hall slope remains negative but decrease in magnitude with increasing gate voltage. In Fig. 5b, the red spheres show the Hall slope in the perturbed region of the graphene, which stays positive over the span − 50$<V_{G}<$+ 17 V, with a small ripple within the span − 5$<V_{G}<$0 V. From + 12$<V_{G}<$+ 25 V, slope changes from positive to negative. Above the gate voltage + 25 V, the slope remains negative but decreases in magnitude. Here, the crossover band for the unperturbed region is narrower than the band for perturbed area. This suggests that the perturbed region could also have become more inhomogeneous after the perturbation.

**Figure 6:**
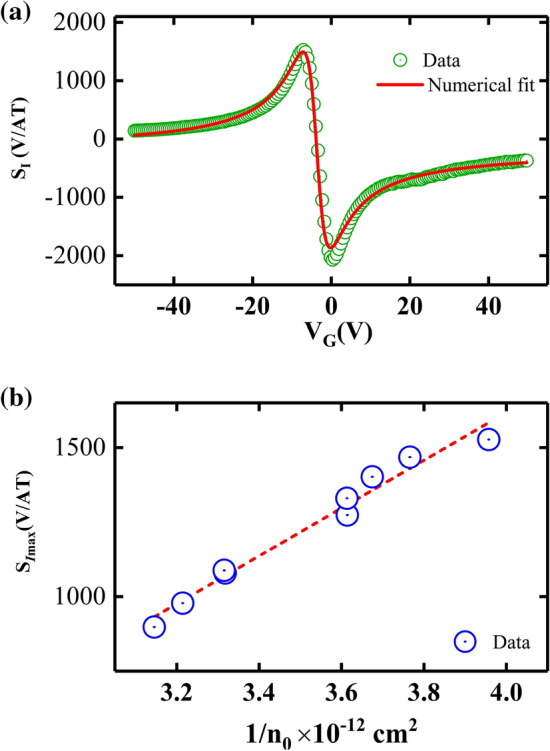
Characteristic curves of the current related sensitivity $S_{I}$ in the unperturbed region of the Hall bar device. (**a**) The $S_{I}$ of the Hall bar device as a function of the gate voltage at a constant magnetic field. Green circles are data and solid red curve is the numerical fit. (**b**) This panel shows that the maximum current sensitivity $S_{Imax}$ is inversely proportional to residual carrier concentration $n_{0}$ in graphene channel. Blue circles are data and dashed red line is a guide to the eye.

As a supplementary result, the current related Hall sensitivity ($S_{I}$) of the unperturbed device as a function of gate voltage and magnetic field is shown in Fig. 6. The current related sensitivity is defined as $S_{I}=V_{xy}/IB=\rho _{xy}/B$^26^. Both types of carriers play an important role near to the charge neutrality points of graphene, not as in unipolar type conventional semiconductors. Thus, we utilized ambipolar transport model to describe the experimental data as suggested in a previous study^27^. The current related sensitivity can be written as Eq. (2).

2 $$\begin{aligned} S_{I}=\frac{r_{H}}{e} \frac{n_{h}\mu _{h}^2 - n_{e}\mu _{e}^2}{(n_{h}\mu _{h} + n_{e}\mu _{e})^2} \end{aligned}$$

Where, *e* is the electron charge, $r_{H}$ is the Hall coefficient, $n_{h}$ and $\mu _{h}$ are density and mobility of hole carriers, and $n_{e}$ and $\mu _{e}$ are density and mobility of electrons. By assuming $\mu _{e}=\mu _{h}$ close to neutrality point, we can derive Eq. (3).

3 $$\begin{aligned} S_{I}=-\frac{r_{H}}{e} \frac{n_{[V_{G}]}}{n_{[V_{G}]}^2+n_{0}^2} \end{aligned}$$

Where, $n_{[V_{G}]}=\alpha (V_{neutrality}-V_{G})$ and $\alpha = 7.2 \times 10^{10} \; \text{cm}^{-2} \; \text{V}^{-1}$. We utilized Eq. (3) to fit the experimental data from unperturbed region in graphene as shown Fig. 6a. We extract the residual carrier concentration $n_{0}$ from the fittings and $n_{0}$ is inversely propositional to maximum current sensitivity as described in Fig. 6b. Here, the maximum current sensitivity is equal to 2635 V/AT which is compatible with previously reported sensitivity values for graphene based devices^27,28^. Note that the reported maximum current related sensitivity values based on graphene devices are higher than conventional Hall sensors fabricated from GaAs/AlGaAs ($\sim 1500$ V/AT ), GaAs ($\sim 200$ V/AT ), and InAs ($\sim 500$ V/AT )^27,29^. However, the Hall sensitivity corresponding to perturbed region of graphene does not obey the above fitting function perhaps because the Hall sensitivity fitting function assumes uniform carrier density and equal electron and hole mobilities, which may not hold true in the perturbed region.

## Summary

We utilized the electrical stressing-voltage-method to create p-n junctions in relatively large area CVD multi-terminal graphene Hall bar devices and examined their transport characteristics. The Hall voltage measurements across the electrical stress-induced neutrality point shows magnetic field dependence not observed in the unstressed device. Further, it appears that the extracted mobility for the unperturbed graphene region is higher than the mobility in perturbed region. The current related maximum sensitivity is 2635 V/AT in unperturbed region of the graphene device. These results help to better understand the creation and behavior of double neutrality points in graphene field effect transistors, in particular, the role of charge carrier characteristics for real world applications based on graphene p-n junctions.

### Supplementary Information

Supplementary Figures.

## Data Availability

Data is provided within the manuscript and in supplementary information files.
