# Supplementary material for: Study of graphene p-n junctions formed by the electrostatic modification of the SiO2 substrate
Source: Sci Rep. 2024 May 28;14:12154. doi: 10.1038/s41598-024-61683-2 (PMC11130296; doi:10.1038/s41598-024-61683-2)
Supplement: Supplementary file 1 — Supplementary Figures. [file 41598_2024_61683_MOESM1_ESM.pdf]

Supplementary: Study of graphene p-n junctions formed by the electrostatic modification of the SiO_2 substrate

Tharanga R. Nanayakkara[1,2], U. Kushan Wijewardena[1,3], Annika Kriisa[1], Ramesh G. Mani[1]

[1]Georgia State University, Atlanta, GA 30303 USA

[2]Brookhaven National Laboratory, Upton, NY 11973 USA

[3]Georgia College and State University, Milledgeville, GA 31061 USA

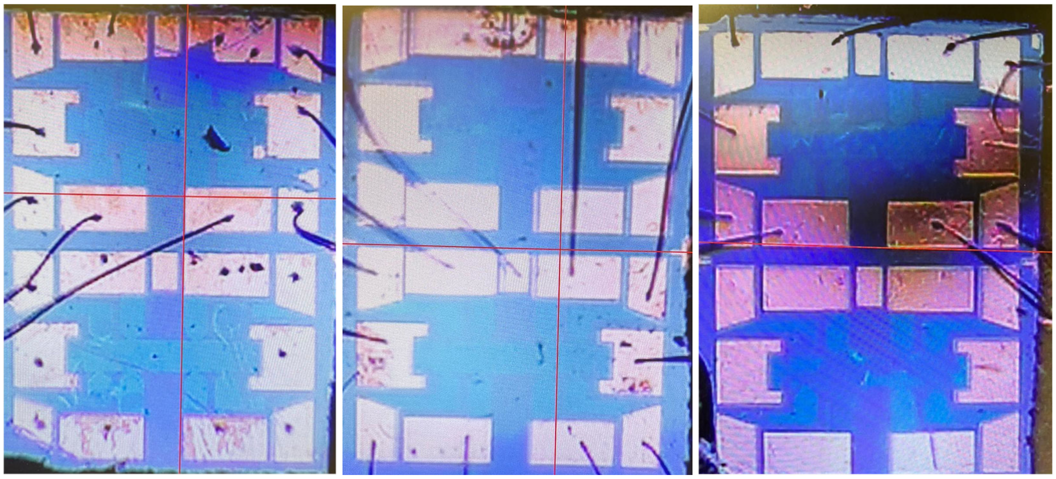

Figure S1) This figure shows photographs of some graphene Hall bar devices fabricated for this study on SiO_2/Si substrates.

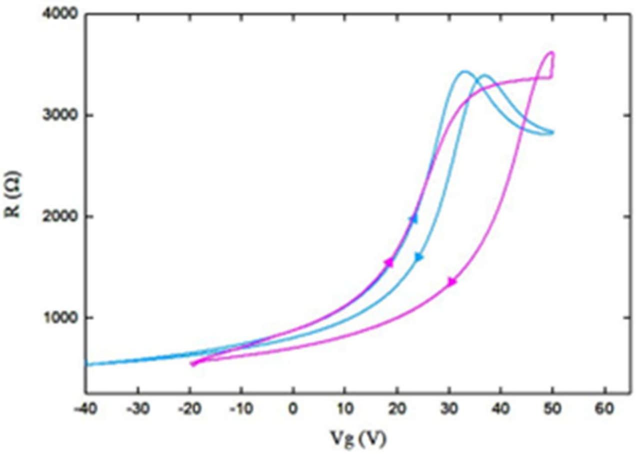

Figure S2: A comparison of the hysteresis in a freshly prepared graphene device with that seen after specimen storage in vacuum. The pink solid line represents the longitudinal resistance as a function of the gate voltage in a freshly prepared device. The light blue line shows the longitudinal resistance as a function of gate voltage after storage under vacuum. Storage in vacuum reduces the hysteresis and shifts the neutrality point towards V_g = 0V.

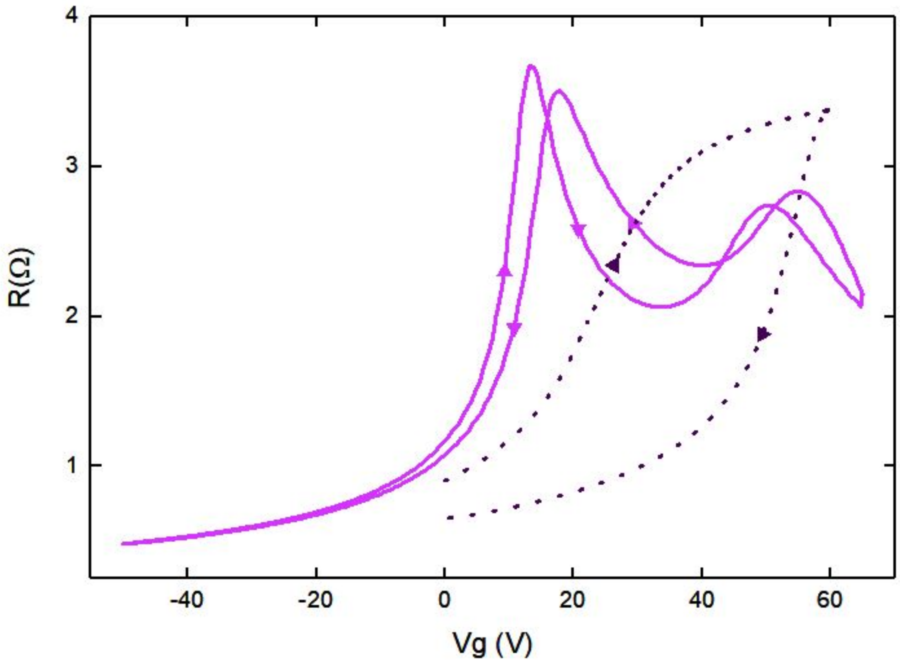

Figure S3: Repeatability of pn-junction formation in the graphene device by applying the electrical stressing technique, after the specimen was thermally cycled from low temperature to ambient conditions. The dashed line shows the longitudinal resistance as a function of gate voltage after warming up the sample to ambient conditions. The solid pink line shows the longitudinal resistance as a function of gate voltage at low temperature after another instance of electrically stressing the device. A p-n junction is realized at V_g between the two peaks in R.
